# Adaptive Frame Structure Design for Sensing-Assisted Downlink Communication in the Vehicle-to-Infrastructure Scenario

**DOI:** 10.3390/s24155061

**Published:** 2024-08-05

**Authors:** Junliang Yao, Ze Wang, Chunli Zhang, Hui Hui

**Affiliations:** Shaanxi Key Laboratory of Complex System Control and Intelligent Information Processing, Xi’an University of Technology, Xi’an 710048, China; yaojunliang@xaut.edu.cn (J.Y.); 13389128038@163.com (Z.W.); huihui@xaut.edu.cn (H.H.)

**Keywords:** V2X, sensing-assisted communication, channel estimation, adaptive frame structure

## Abstract

Vehicle-to-everything (V2X) is considered a key factor in driving the future development of intelligent transport, which requires high-quality communication and fast sensing of vehicle information in high-speed mobile scenarios. However, high-speed mobility makes the wireless channel change rapidly, which requires frequent channel estimation and channel feedback between a vehicle and the roadside unit (RSU), resulting in an increase in communication overhead. At the same time, the high maneuverability of vehicles leads to frequent switching and misalignment of communication beams, so the RSU must have better beam prediction and tracking capabilities. To address this problem, this paper proposes an adaptive frame structure design scheme for sensing-assisted downlink (DL) communication. The basic idea of the scheme involves analyzing the communication model during the vehicle’s movement. This analysis aims to establish a theoretical relationship between the Symbol Error Rate (SER) and the following two key factors: the vehicle’s starting position and the distance it travels across. Subsequently, the scheme leverages the vehicle’s position data, as detected by the RSU, to calculate the real-time SER for DL communication. The SER threshold is set based on the requirements of DL communication. If the real-time SER is below this threshold, channel estimation becomes unnecessary. This decreases the frequency of channel estimation and frees up time and frequency resources that would otherwise be occupied by channel estimation processes within the frame structure. The design of an adaptive frame structure, as detailed in the above scheme, is presented. Furthermore, the performance of the proposed method is analyzed and compared with that of the traditional communication protocol frame structure and the beam prediction-based frame structure. The simulation results indicate that the communication throughput of the proposed method can be improved by up to 6% compared with the traditional communication protocol frame structure while maintaining SER performance.

## 1. Introduction

Fifth-generation (5G) mobile communication technology enables a leap in communications from simple voice/text services and data services to the Internet of Everything, facilitating continued growth and innovation in areas such as smart transport, healthcare, smart manufacturing, drones, and more [1,2]. V2X is a typical application of intelligent transport in high-speed mobile scenarios and is considered a key enabler of future intelligent transport, which is expected to make the transport industry more efficient and safer on the road to a large extent [3]. In order to achieve this goal, vehicles need to have the ability to simultaneously sense information about their surroundings and exchange information with roadside units and other vehicles [4]. This necessitates that V2X possesses not only enhanced communication capabilities but also accurate sensing functions to meet a wide range of demands [5]. In this context, the research on communication-aware fusion and resource allocation strategies in the V2X scenario has received great attention [6,7].

In the V2X scenario, Ref. [8] proposes utilizing an Orthogonal Frequency Division Multiplexing (OFDM) signal for vehicle positioning while ensuring communication efficiency. In Ref. [9], the Positioning Reference Signal (PRS) of 5G mobile communication is utilized to achieve sensing, communication, and positioning capabilities. The study in Ref. [10] employs reference signals in 5G for sensing and optimizes transmission power allocation. This optimization is subject to the performance of the communication channel and the radar sensing ambiguity function. In Refs. [11,12], resource allocation in both the time domain and frequency domain is conducted with the aim of enhancing channel capacity while ensuring that the requirements of sensing accuracy are met. In Ref. [13], the sensing performance of V2X communication is analyzed, considering the existence of interference among vehicles. Additionally, the impact of the system’s resource allocation strategy on sensing performance is discussed. In Ref. [14], the impact of communication parameters such as bandwidth, modulation and coding schemes, and packet size on sensing performance is analyzed in terms of detection capability and parameter estimation error, while the impact of resource allocation strategies on communication and sensing performance is considered.

Vehicle-to-Infrastructure (V2I) is an important subset of V2X applications. In the V2I scenario, RSUs are permanently installed along roadsides and equipped with robust capabilities for data processing and resource management [15]. These RSUs sense the surrounding environment and interact with vehicles, providing them with real-time information about current road conditions. This enables timely adjustments to driving strategies and improves communication efficiency. For V2I communication to ensure traffic safety and effectiveness, it must have a high data rate and low transmission delay; otherwise, it could introduce traffic safety issues. Considering that RSUs possess functions of communication, sensing, and data processing, beam prediction and tracking can be effectively executed on this basis. Furthermore, the comprehensive system performance is significantly enhanced through the optimization of resource allocation [16]. A joint task offloading and resource allocation scheme is proposed, which can minimize the total task processing delay for all vehicles through task scheduling and channel allocation for both the vehicle and the RSU [17].

In addition, effective beam tracking can ensure the establishment of a stable communication link and improve communication quality in the V2I scenario [18]. Currently, a variety of methods exist for beam tracking and prediction. In Ref. [19], a beam tracking scheme based on the variable step beam is proposed. This scheme addresses the challenges of user mobility in beam tracking by utilizing a fraction of the beam energy. It does so without altering the direction or width of the current communication beam. The majority of the beam energy is dedicated to sustaining the quality of communication. Furthermore, the paper introduces an Angle of Departure (AoD) estimation algorithm using Bayesian posterior probability. This algorithm employs Gaussian process regression to refine the beam’s direction and breadth for the subsequent frame. In Ref. [20], an analysis of a beam tracking scheme for extremely large-scale multiple-input–multiple-output communication systems is presented. The scheme introduces a near-field beam tracking approach that leverages a dynamic model to anticipate channel variations. By incorporating the user’s location estimated through extended Kalman filtering and the kinematic model, it effectively tracks and forecasts near-field channel variations, thus minimizing the computational burden associated with beam tracking. In Ref. [21], a deep neural network-based novel downlink beam prediction scheme is introduced. The proposed neural network utilizes an adjustable feature fusion learning mechanism to embed user location data into CSI, which aims to reduce the beam training overhead. Gonzalez proposes a scheme for communication in a V2I scenario using a millimeter wave, which can enhance both the data transmission rate and the sensing accuracy [22]. Meanwhile, large-scale antenna arrays in millimeter-wave communication can provide high beam gain and reduce interference among vehicles [23]. On this foundation, beam tracking methods based on codebook design and Kalman filtering are proposed in Refs. [24,25], with the aim of establishing a stable communication link between the receiver and the transmitter.

However, in the high-speed movement scenario, the high maneuverability of vehicles in V2I communications can lead to frequent switching and misalignment of communication beams, potentially causing interruptions in the communication link [18]. This necessitates frequent coordination and feedback between the RSU and the vehicle, resulting in a significant increase in communication overhead. To address the aforementioned issues, beam tracking assisted by sensing is considered as an effective solution. This method of sensing-assisted beam tracking eliminates the need for dedicated DL pilots, aligns the beams while simultaneously performing environmental sensing, and can significantly reduce channel estimation overhead, offering broader prospects [26,27].

Numerous studies have utilized sensing-assisted communication to reduce the beam tracking overhead in the V2I scenario. In Ref. [28], a vehicle tracking algorithm based on extended Kalman filtering is proposed, which improves the stability of the communication link by predicting the beam and reduces the beam tracking overhead simultaneously. In Ref. [29], a sensing-assisted beamforming scheme that takes into account vehicle geometry is investigated. Initially, the entire vehicle is covered by a wide beam. Subsequently, the vehicle’s position is tracked using the extended Kalman filtering method. Finally, the communication process is completed with a narrow beam, enhancing communication efficiency. In Ref. [30], a curvilinear coordinate system is combined with a multi-model extended Kalman filtering framework to model complex road geometries. This approach accurately tracks a vehicle’s motion on complex roads based on the sensing function. Additionally, a sensing-assisted beam tracking scheme applicable to roads of arbitrary shapes is proposed. This scheme aims to improve vehicle tracking accuracy and enhance communication quality in the V2I scenario. In Refs. [31,32], a beam prediction scheme based on sensing-assisted communication is proposed. In this scheme, the RSU estimates and predicts the dynamic parameters of a vehicle using signal echoes and achieves beam tracking based on the vehicle’s perception. This method can significantly reduce the beam training overhead and improve the communication efficiency between the RSU and the vehicle, compared with the traditional feedback-based beam tracking method.

The research scheme mentioned in the previous paragraph involves the design of the beam for the RSU, which is aided by sensing capabilities. This assistance minimizes the need for frequent information feedback with the vehicle, thereby reducing the beam tracking overhead in the communication process. However, the vehicle is unable to predict beam changes during DL decoding. The delay and Doppler shift that occur during the vehicle’s movement can lead to an increase in the Symbol Error Rate (SER) in downlink (DL) communication. DL refers to the transmission path from the RSU to the vehicle, and the SER indicates the number of erroneous symbols received in a transmission relative to the total number of transmitted symbols. Consequently, it becomes necessary to re-estimate the channel to facilitate decoding. Considering the time-varying channel and the high overhead of periodic channel estimation in the V2I scenario, this paper proposes a sensing-assisted adaptive frame structure design scheme for DL communication. Given that the wireless channel fading in the V2I scenario is primarily determined by the relative positions of the vehicle and the RSU [33], when the vehicle’s movement is within a certain range, the DL channel exhibits a high time correlation. The channel state information (CSI) from the previous position can be utilized to complete the Fcommunication process. This approach reduces the frequency of channel estimation during the vehicle’s movement and, consequently, lowers the communication overhead. When the vehicle moves beyond a certain distance, the channel correlation diminishes, necessitating a new channel estimation. To illustrate the impact of channel temporal correlation on communication performance, the theoretical analysis presents the communication SER as a function of lane shape, vehicle starting position, and vehicle moving distance. Once the RSU acquires the vehicle motion parameters through sensing, it can compute the real-time SER and decide whether to incorporate pilots in the current frame based on the communication performance requirements.

To verify the performance of the proposed method, experiments can be conducted using simulators. Among the existing simulators, CARLA provides a comprehensive suite of functionalities, including weather conditions, digital assets, and traffic scenario management. These capabilities facilitate the configuration of sensor suites and environmental conditions [34]. CAVISE can simulate traffic flows and vehicle behaviors and is capable of modeling a variety of environmental conditions and road conditions to evaluate their effects on the performance of vehicular communication systems [35]. In V2X scenarios, the Artery framework is utilized for the development of network and signal propagation models [35]. SUMO, an open-source traffic simulator, is well-suited for managing large-scale traffic flows. Its functionality is particularly useful for simulating vehicle communications and traffic management strategies [36]. OpenCDA is an advanced platform that contains self-driving modules for sensing, computation, actuation, and communication. However, OpenCDA only provides a basic representation of vehicular communication [37]. The aforementioned simulators have difficulties in supporting the modification and validation of underlying communication algorithms, including signal modulation, demodulation, and frame structure design. Consequently, by leveraging the existing models in MATLAB, we developed a design for an adaptive frame structure scheme. Looking ahead, once the compatibility issues between MATLAB and other simulators like CARLA, CAVISE, and Artery are resolved, it will become feasible to implement the proposed scheme across the existing simulation platforms.

The main contributions of this paper are as follows.

(1) In the V2I scenario, the RSU is assumed to have the capability to acquire vehicle position and speed through sensing. The theoretical analysis outlines the function relationship between the communication SER and various initial positions of the vehicle, as well as different moving distances.
(2) A sensing-assisted communication adaptive frame structure design is proposed. Specifically, a communication SER threshold is established. If the communication SER exceeds the threshold value during vehicle movement, the RSU must retransmit the pilots for channel estimation; otherwise, the pilots are not transmitted. The proposed scheme can adaptively adjust the transmission interval of pilots within the frame structure based on the vehicle’s initial position, the vehicle’s moving speed, and the communication SER requirements.
(3) The SER and throughput performance of three frame structures are comparatively analyzed in both straight and curved path scenarios. These structures include the traditional communication protocol frame structure, the existing beam prediction-based frame structure, and the proposed sensing-assisted adaptive frame structure.

This paper is organized as follows: Section 2 introduces the transmit signal, channel model, and receive signal within the V2I DL communication system model; Section 3 conducts a theoretical analysis of the DL communication processes for both straight and curved path scenarios, proposing a sensing-assisted communication adaptive frame structure design scheme; Section 4 presents the simulation experiments and result analyses concerning the SER and throughput; and finally, Section 5 provides a summary of the entire paper.

## 2. V2I Downlink Communication System Model

**Figure 1:**
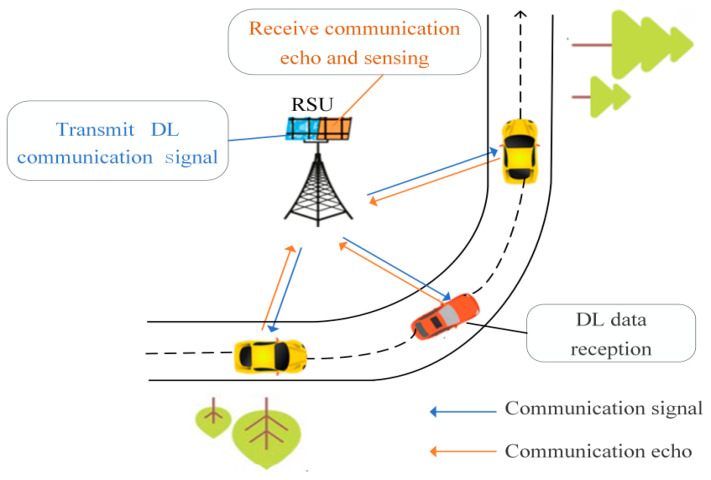
System scenario model.

In this paper, we consider the DL communication process of the V2I scenario and the system model depicted in Figure 1. The RSU is equipped with a uniform equally spaced surface array, comprising independent transmit and receive arrays. The dimension of the transmitting antenna array is $P_{t}\times Q_{t}$, and the dimension of the receiving antenna array is $P_{r}\times Q_{r}$. The vehicle is equipped with a single antenna and is assumed to be a point target. During the DL communication, the RSU sends signals to the vehicle and receives echo signals from the direction of the vehicle for sensing signal processing. The vehicle receives the DL communication signals from the RSU for the purposes of synchronization and channel estimation and subsequently uses them to demodulate and retrieve the final data.

### 2.1. Transmit Signal

In the V2I scenario considered in this paper, DL communication is conducted using the OFDM signal, and the transmitted symbols are illustrated in the following equation:

(1) $$s(t)=\sum_{m=0}^{M_{s}}\sum_{n=0}^{N_{c}}\sqrt{p_{t}}u_{n,m}e^{j2\pi \left(f_{c}+n\Delta f\right)t}Rect\left(\frac{t-mT_{s}}{T_{s}}\right)$$

where $M_{s}$ is OFDM symbol number, $N_{c}$ is subcarrier number, $p_{t}$ is the symbol transmit power, $u_{n,m}$ is the OFDM baseband symbol of the *n*th subcarrier and the *m*th symbol, $f_{c}$ denotes the carrier frequency, and Δf and $T_{s}$ are the subcarrier interval and the length of OFDM symbol time. Rect(*t*/*T_s_*) is the rectangular function, which is equal to 1 for *t* < *T_s_* and 0, otherwise.

### 2.2. Channel Model

(1) DL communication channel model.

The channel coefficients for the DL communication channel on the *n*th subcarrier and the *m*th OFDM symbol are expressed as follows [38,39]:

(2) $$H_{C,n,m}=\sum_{l=0}^{L-1}b_{C,l}e^{j2\pi \left(f_{C,l}\right)mT_{s}}e^{-j2\pi n\Delta f\left(\tau_{C,l}\right)}\times a^{T}\left(\theta_{TX,l}\right)$$

where *L* is the number of multipaths and $b_{C,l}$ is the channel fading coefficient of the *l*th path. Specifically, for the Line-of-Sight (LoS) path, the channel fading coefficient is $b_{C,0}=\lambda /\left(4\pi d_{0}\right)$, where $d_{0}$ is the distance from TX to RX. The *l*th multipath (*l* > 0) fading coefficient is expressed as follows:

(3) $$b_{C,l}=\sqrt{\frac{\lambda^{2}}{{\left(4\pi \right)}^{3}d_{l,T}^{2}d_{l,R}^{2}}}\beta_{C,l}$$

where λ is the wavelength and $d_{l,T}$ and $d_{l,R}$ are the distances from TX to the scatterer and from the scatterer to RX, respectively. The reflection fading $\beta_{C,l}\sim CN\left(0,\sigma_{\beta ,l}^{2}\right)$ and $\sigma_{\beta ,l}^{2}$ can be regarded as the parameter related to the reflecting surface, which can be considered to change slowly if the reflector remains unchanged. $f_{C,l}$ is the Doppler shift of the *l*th path and $\tau_{C,l}=\left(d_{l,T}+d_{l,R}\right)/c$ is the time delay of the *l*th path. *c* is the propagation speed of the electromagnetic wave in vacuum. The column vector $a\left(\theta_{TX,l}\right)$ denotes the steering vector for transmission, and the (*p*,*q*)th element is denoted as follows:

(4) $$a_{p,q}\left(\theta_{TX,l}\right)=\exp\left[-j\frac{2\pi }{\lambda }d_{a}\left(p\cos\left(\theta_{TX,l}\right)+q\sin\left(\theta_{TX,l}\right)\right)\right]$$

where $d_{a}$ is the antenna interval and $\theta_{TX,l}$ is the horizontal angle of the transmitter. During DL communication, there is only the steering vector for transmission because the vehicle has a single antenna. Considering that the reflective path channel fading is much larger than the LoS path in the V2I scenario, only the LoS path is considered in this paper.

(2) Sensing channel model.

The channel coefficients on the *n*th subcarrier of the sensing channel and on the *m*th OFDM symbol are expressed as follows [38,39]:

(5) $$H_{S,n,m}=\sum_{l=0}^{L-1}b_{S,l}e^{j2\pi \left(f_{S,l}\right)mT_{s}}e^{-j2\pi n\Delta f\left(\tau_{S,l}\right)}\times a\left(\theta_{RX,l}\right)a^{T}\left(\theta_{TX,l}\right)$$

where $b_{S,l}$ is the channel fading coefficient for the *l*th multipath (*l* = 0 for the LoS path) and is expressed as follows:

(6) $$b_{S,l}=\sqrt{\frac{\lambda^{2}}{{\left(4\pi \right)}^{3}d_{l}^{4}}}\beta_{S,l}$$

where the reflection fading $\beta_{S,l}\sim CN\left(0,\sigma_{\beta ,l}^{2}\right)$ and $\sigma_{\beta ,l}^{2}$ can be regarded as a parameter related to the reflecting surface, which can be considered to change slowly if the reflector remains unchanged. $d_{l}$ is the distance from the antenna array to the reflector. $f_{S,l}=2v_{l}/\lambda$ is the Doppler shift of the *l*th echo path, where $v_{l}$ is the radial relative velocity. $\tau_{S,l}=2d_{l}/c$ denotes the round-trip time delay of the *l*th echo path. $\theta_{RX,l}$ denotes the horizontal angle of the receiver, and $a\left(\theta_{RX,l}\right)$ is the column vector of $P_{r}Q_{r}\times 1$, whose elemental representation is similar to that of $a\left(\theta_{TX,l}\right)$.

### 2.3. Receive Signal

The received signal at the vehicle on the *n*th subcarrier and the *m*th OFDM symbol is expressed as follows:

(7) $$y_{C,n,m}=\sqrt{p_{t}}H_{C,n,m}w_{TX}u_{n,m}+n_{n,m}$$

where $w_{TX}$ denotes the DL transmit beamforming vector and $n_{n,m}$ denotes the additive Gaussian white noise. The sensing receive signal on the *n*th subcarrier and on the *m*th OFDM symbol at the receiver side of the RSU is expressed as follows:

(8) $$y_{S,n,m}=\sqrt{p_{t}}H_{S,n,m}w_{TX}u_{n,m}+n_{n,m}$$

## 3. Adaptive Frame Structure Design

**Figure 2:**
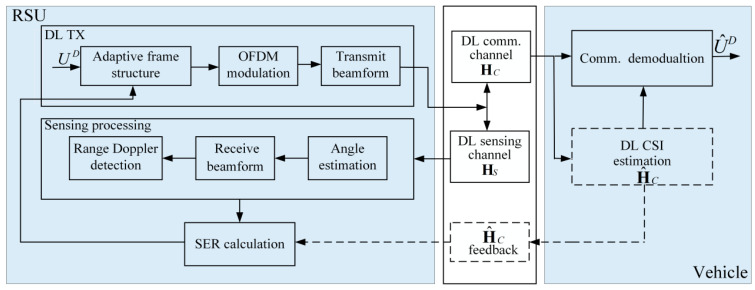
The DL communication process.

The DL communication process of the proposed adaptive frame structure is illustrated in Figure 2. During the initial communication, the RSU sends a DL signal $U^{D}$ to the vehicle. The pilots embedded within the signal frame are employed for channel estimation purposes. All symbols within the signal frame can be used for sensing signal processing, which facilitates the extraction of vehicle motion parameters. The DL communication signal, after undergoing OFDM modulation and beamforming, is transmitted to the vehicle via the DL communication channel. The vehicle receives the DL communication frame from the RSU, utilizes the pilots to estimate the CSI, and then demodulates the information symbols ${\hat{U}}^{D}$. Concurrently, it carries out the Uplink (UL) CSI feedback. Upon receiving the signal echo, the RSU utilizes the echo signal for sensing to determine the motion parameters of the vehicle [9].

Utilizing vehicle position, moving distance, and other parameters, along with UL channel feedback, the DL communication SER for the current downlink communication frame is calculated. This calculated SER serves as the basis for deciding the transmission of channel estimation pilots in the subsequent frame, which is part of the adaptive frame structure module. If the SER is below a preset threshold, the RSU does not need to send DL pilots, and the vehicle can omit channel estimation. If not, the channel must be re-estimated. Consequently, the proposed method can not only effectively reduce the frequency of pilot transmission but also prevent frequent feedback exchanges between the RSU and the vehicle, thereby reducing the communication overhead.

Based on the above content, a DL communication model is designed to improve the efficiency of information exchange between the RSU and vehicles within the V2I scenario. The model is categorized under the Digital Information layer (Layer 6). This layer is primarily focused on the information exchange among vehicles, infrastructure, and other objects via digital signals in V2X environments [40,41].

### 3.1. SER Analysis of DL Communication during Vehicle Movement

In the high-speed V2I scenario, there are strong LoS paths between the RSU and the vehicle, with fewer scattering paths in their vicinity. The channel coefficients for the *m*th OFDM symbol and the *n*th subcarrier are considered in the LoS channel DL communication process:

(9) $$H_{C,n,m}={b_{C,0}e}^{j2\pi \left(f_{C,0}\right)mT_{s}}e^{-j2\pi n\Delta f\left(\tau_{C,0}\right)}a^{T}\left(\theta_{0,0}\right)$$

where $\theta_{0,0}$ denotes the initial angle of the vehicle and the subscripts (0,0) denote the LoS scenario and the initial position of the vehicle, respectively. Consequently, the received signal for DL communication in Equation (7) can be expressed as follows:

(10) $$\begin{array}{l} y_{C,n,m}=\sqrt{p_{t}}h_{C,n,m}a^{T}\left(\theta_{0,0}\right)w_{TX}\left(\theta_{0,0}\right)u_{n,m}+n_{n,m}\\ \;\;\;=\sqrt{p_{t}}h_{C,n,m}m_{v}u_{n,m}+n_{n,m}\\ \;\;\;=x_{n,m}+n_{n,m} \end{array}$$

where $h_{C,n,m}={b_{C,0}e}^{j2\pi \left(f_{C,0}\right)mT_{s}}e^{-j2\pi n\Delta f\left(\tau_{C,0}\right)}$ and $w_{TX}\left(\theta_{0,0}\right)$ is the column vector with dimension $P_{t}Q_{t}\times 1$. $m_{v}=a^{T}\left(\theta_{0,0}\right)w_{TX}\left(\theta_{0,0}\right)$ denotes the beam matching gain; if the two angles are identical, the beam directivity becomes stronger, which can effectively enhance the DL receiving power and improve communication quality. Conversely, if the two angles differ, it will result in a degradation of communication quality. The single-carrier information power in the received signal is expressed as follows:

(11) $$\begin{array}{l} E\left[{\left|x_{n,m}\right|}^{2}\right]=E\left[p_{t}{\left|h_{C,n,m}\right|}^{2}{\left|m_{v}\right|}^{2}{\left|u_{n,m}\right|}^{2}\right]\\ \;\;\;\;\;\;=p_{t}{\left|b_{C,0}\right|}^{2}{\left|e^{j2\pi \left(f_{C,0}\right)mT_{s}}e^{-j2\pi n\Delta f\left(\tau_{C,0}\right)}\right|}^{2}{\left|m_{v}\right|}^{2}E{\left[\left|u_{n,m}\right|\right]}^{2} \end{array}$$

where *E*[*x*] denotes the expectation of the variable *x*. Assuming that the transmitted symbol power satisfies $E\left[{\left|u_{n,m}\right|}^{2}\right]=1$, and the noise power is $E\left[{\left|n_{n,m}\right|}^{2}\right]=\sigma_{N}^{2}$, the Doppler shift of the LoS path $f_{C,0}$ and the time delay $\tau_{C,0}$ will alter the phase of the received signal, thereby affecting the communication demodulation performance. However, the impact on demodulation can be mitigated in practice by estimating the phase deviation and compensating for it. Parameter estimation errors and variations due to continuous vehicle motion will be discussed subsequently. Consequently, the single-carrier Signal-to-Noise Ratio (SNR) of the received signal is expressed as follows:

(12) $$SNR=\frac{E\left[{\left|x_{n,m}\right|}^{2}\right]}{E\left[{\left|n_{n,m}\right|}^{2}\right]}=\frac{p_{t}{\left|b_{C,0}\right|}^{2}{\left|m_{v}\right|}^{2}E\left[{\left|u_{n,m}\right|}^{2}\right]}{E\left[{\left|n_{n,m}\right|}^{2}\right]}=\frac{p_{t}{\left|b_{C,0}\right|}^{2}{\left|m_{v}\right|}^{2}}{\sigma_{N}^{2}}$$

During the vehicle’s movement, the position corresponding to different frames in the DL will vary and, consequently, the associated channel fading coefficients will also fluctuate. Subsequently, $b_{C,0}$ is analyzed for both straight and curved path scenarios.

(1) Straight path scenario.

**Figure 3:**
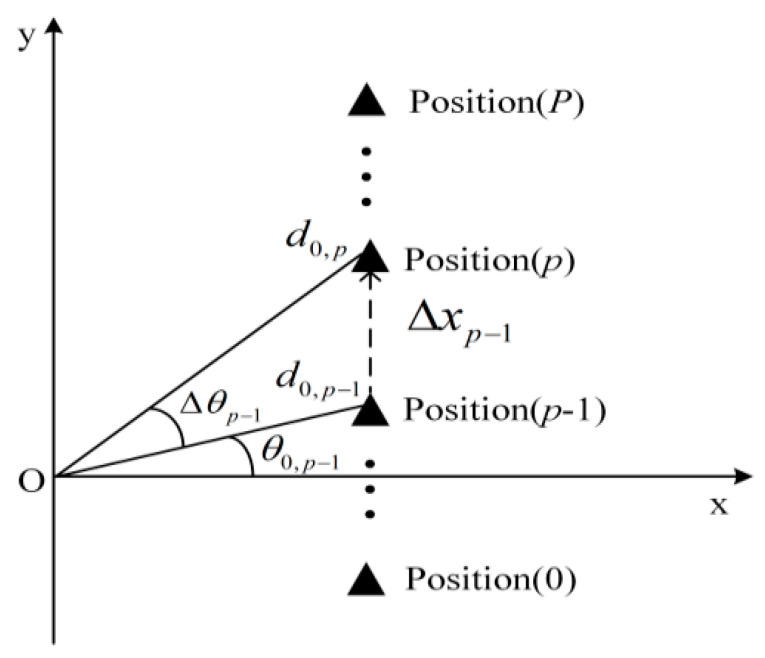
Schematic diagram of straight path scenario.

In the straight path scenario depicted in Figure 3, the origin O represents the RSU, and the black triangle represents the vehicle. When the vehicle is at position *p*, its distance from the RSU is $d_{0,p}$, and the angle is $\theta_{0,p}$, where *p* = 0, 1, 2, …, *P* denotes the vehicle’s position. $\Delta x_{p-1}$ denotes the distance between position *p* − 1 and position *p*, and $\Delta \theta_{p-1}$ denotes the corresponding change in angle.

From the diagram above, it can be observed that the vehicle moves a distance of:

(13) $$\Delta x_{p-1}=d_{0,p}\sin(\theta_{0,p-1}+\Delta \theta_{p-1})-d_{0,p-1}\sin\theta_{0,p-1}$$

The updated distance from the vehicle to the RSU is expressed as follows:

(14) $$d_{0,p}=\left(d_{0,p-1}\sin\theta_{0,p-1}+\Delta x_{p-1}\right)/\sin(\theta_{0,p-1}+\Delta \theta_{p-1})$$

By substituting $d_{0}=d_{0,p}$ into $\left|b_{C,0}\right|=\lambda /\left(4\pi d_{0}\right)$, the channel fading coefficient for the vehicle at position *p* is obtained as follows:

(15) $$\begin{array}{l} \left|b_{C,0}^{p}\right|=\lambda /\left(4\pi \left(\left(d_{0,p-1}\sin\theta_{0,p-1}+\Delta x_{p-1}\right)/\sin(\theta_{0,p-1}+\Delta \theta_{p-1})\right)\right)\\ \;\;\;\;=\left(\lambda \sin(\theta_{0,p-1}+\Delta \theta_{p-1})\right)/\left(4\pi (d_{0,p-1}\sin\theta_{0,p-1}+\Delta x_{p-1})\right) \end{array}$$

(2) Curved path scenario.

**Figure 4:**
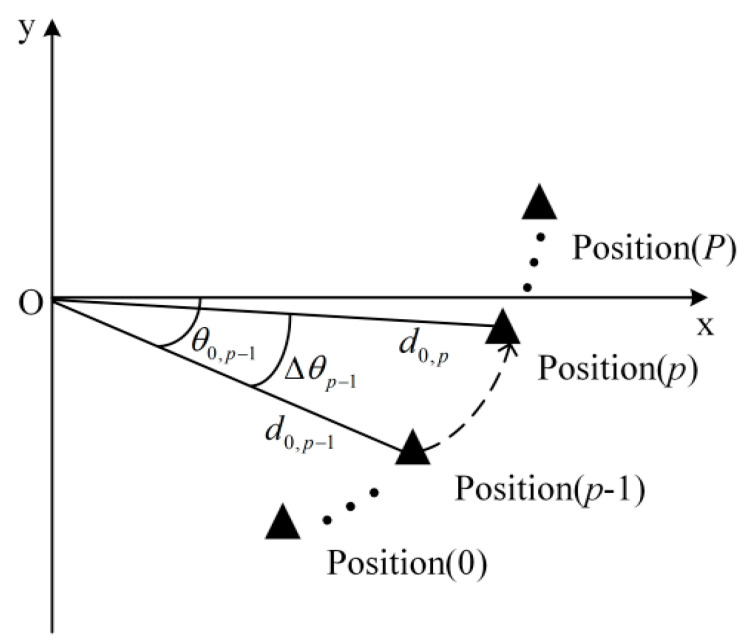
Schematic diagram of curved path scenario.

In the curved path scenario illustrated in Figure 4, the change in distance between the vehicle and the RSU as the vehicle transitions from the depicted position *p*−1 to position *p* is denoted by $\Delta d_{p-1}$, and the corresponding change in the vehicle’s angle is denoted by $\Delta \theta_{p-1}$. At position *p,* the distance between the vehicle and the RSU is $d_{0,p}=d_{0,p-1}+\Delta d_{p-1}$, and the vehicle’s angle is $\theta_{0,p}=\theta_{0,p-1}+\Delta \theta_{p-1}$. By substituting $d_{0}=d_{0,p}$ into $\left|b_{C,0}\right|=\lambda /\left(4\pi d_{0}\right)$, the channel fading coefficient for the vehicle at position *p* is calculated as follows:

(16) $$\left|b_{C,0}^{p}\right|=\frac{\lambda }{4\pi \left(d_{0,p-1}+\Delta d_{p-1}\right)}$$

In this paper, we analyze the scenario where the vehicle conducts channel estimation solely at the initial position, with no further channel estimation at subsequent positions. The initial CSI obtained is then utilized to decode the information. Upon reaching position *p*, the vehicle receives the signal in the following manner:

(17) $$y_{C,n,m}=\sqrt{p_{t}}H_{C,n,m}^{p}w_{TX}u_{n,m}+n_{n,m}$$

where $H_{C,n,m}^{p}=b_{C,0}^{p}e^{j2\pi \left(f_{C,0}^{p}\right)mT_{s}}e^{-j2\pi n\Delta f\left(\tau_{C,0}^{p}\right)}a^{T}\left(\theta_{0,p}\right)$ denotes the CSI when the vehicle moves to the *p*th position and $f_{C,0}^{p}$ and $\tau_{C,0}^{p}$ denote the Doppler shift and time delay, respectively. The CSI from the initial position is utilized to complete the communication process as the vehicle moves to the *p*th position along both straight and curved path scenarios. The data obtained by demodulation of the vehicle’s signal at the *p*th position, corresponding to the receiver, can be expressed by the following equation:

(18) $$\begin{array}{l} R_{C,n,m}=y_{C,n,m}/{\hat{H}}_{C,n,m}^{0}\\ \;\;\;\;=\frac{\sqrt{p_{t}}H_{C,n,m}^{p}w_{TX}u_{n,m}+n_{n,m}}{{\hat{H}}_{C,n,m}^{0}}\\ \;\;\;\;=\frac{\sqrt{p_{t}}b_{C,0}^{p}e^{j2\pi \left(f_{C,0}^{p}\right)mT_{s}}e^{-j2\pi n\Delta f\left(\tau_{C,0}^{p}\right)}m_{v}u_{n,m}+n_{n,m}}{{\hat{b}}_{C,0}^{0}e^{j2\pi \left({\hat{f}}_{C,0}^{0}\right)mT_{s}}e^{-j2\pi n\Delta f\left({\hat{\tau }}_{C,0}^{0}\right)}} \end{array}$$

where ${\hat{H}}_{C,n,m}^{0}$ denotes the estimated value of the DL CSI at the initial position, and the Doppler shift ${\hat{f}}_{C,0}^{0}$ and time delay ${\hat{\tau }}_{C,0}^{0}$ of the vehicle at the initial position are included.

As the distance, speed, and angle continue to change during the movement of the vehicle, leading to changes in the channel environment, the true channel CSI at position *p*(*p* ≠ 0) is $H_{C,n,m}^{p}$. When using ${\hat{H}}_{C,n,m}^{0}$ for message decoding, the difference between $H_{C,n,m}^{p}$ and ${\hat{H}}_{C,n,m}^{0}$ results in a higher DL communication SER. The RSU, by sensing the distance, speed, and angle of the vehicle in real time, can perform beam alignment to enhance the DL receive power, i.e., maximizing $m_{v}$ in Equation (18). However, the variation in the channel fading coefficient $b_{C,0}^{p}$, Doppler shift $f_{C,0}^{p}$, and time delay $\tau_{C,0}^{p}$ during vehicle movement will lead to a continuous increase in the SER in DL communication. The RSU can compensate for the phase error caused by the Doppler shift and time delay by sensing the vehicle’s distance and speed in real time. Thus, Equation (18) can be further expressed as:

(19) $$R_{C,n,m}=\sqrt{p_{t}}b_{C,0}^{p}/{\hat{b}}_{C,0}^{0}m_{v}u_{n,m}+n_{n,m}$$

Then, the SNR of the demodulated signal can be further expressed as:

(20) $$S_{e}=E\left[\frac{{\left|R_{C,n,m}\right|}^{2}}{{\left|n_{n,m}\right|}^{2}}=\frac{{p_{t}\left|b_{C,0}^{p}/{\hat{b}}_{C,0}^{0}\right|}^{2}{\left|m_{v}\right|}^{2}}{\sigma_{N}^{2}}\right]$$

The theoretical SER for DL communication during vehicle movement can be calculated by the following equation [42]:

(21) $$\begin{array}{l} P_{e}=2Q(\sqrt{S_{e}})\\ \;=2Q(\sqrt{\frac{{p_{t}\left|b_{C,0}^{p}/{\hat{b}}_{C,0}^{0}\right|}^{2}{\left|m_{v}\right|}^{2}}{\sigma_{N}^{2}}}) \end{array}$$

**Figure 5:**
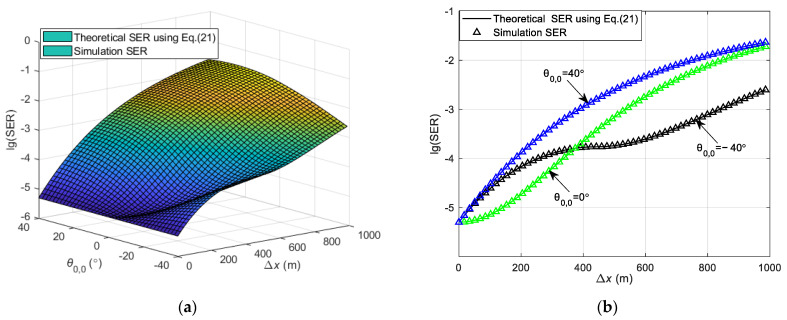
Theoretical and simulation SER of DL communication in the straight path scenario. (**a**) The variation in the SER with the initial position of the vehicle and the moving distance in the straight path scenario. (**b**) The theoretical and simulation SER at the initial positions of the vehicle at $\theta_{0,0}$ = −40°, 0°, and 40°.

**Figure 6:**
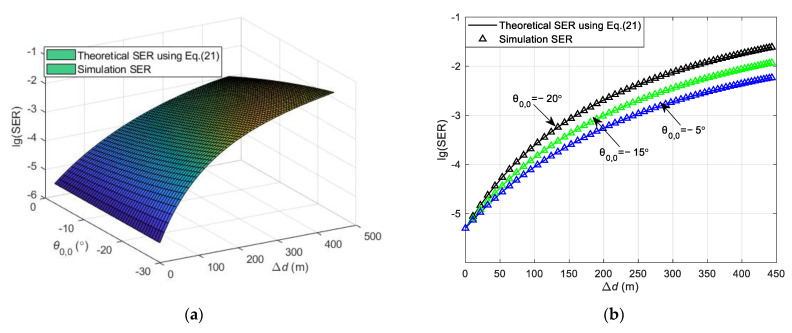
Theoretical and simulation SER of DL communication in the curved path scenario. (**a**) The variation in the SER with the initial position of the vehicle, the distance between the vehicle, and the RSU in the curved path scenario. (**b**) The theoretical and simulation SER at the initial positions of the vehicle at $\theta_{0,0}$ = −20°, −15°, and −5°.

The SER obtained using Equation (21) in both straight and curved paths is depicted in Figure 5 and Figure 6. The SER during the simulation process is defined as the ratio of the number of erroneous symbols at the receiver to the total number of transmitted symbols. This is in contrast to the theoretical SER $P_{e}$, which is derived and calculated using Equation (21). Both represent the SER in the DL communication process. During the simulation, the vehicle’s velocity is kept at a consistent speed, and the vehicle starts moving from a variety of initial positions at different angles relative to the RSU. Channel estimation is conducted as the vehicle begins its movement from an initial position. The SER at this position is determined using Equation (21). Upon arriving at a new location, channel estimation is not re-performed; instead, the CSI obtained from the initial position is utilized to carry out the communication and to determine the SER for that specific location. Consequently, the overall SER for the vehicle throughout its movement trajectory is determined. The carrier frequency $f_{c}$ = 6 GHz, with a subcarrier interval Δf = 30 kHz, and the system employs QPSK modulation. The number of OFDM symbols $M_{s}$ = 14, and the total number of subcarriers $N_{c}$ = 1680, with an OFDM symbol duration $T_{s}$ = 0.0025 s. The antenna interval is configured to be half of the wavelength, and the transmitter antenna array size for the RSU is $P_{t}\times Q_{t}$ = 8 × 8. All results are averaged from 5000 independent Monte Carlo simulations.

Figure 5 illustrates the variation in the DL communication SER with the initial position of the vehicle and the moving distance in the straight path scenario at SNR = 12 dB. $\theta_{0,0}$ presents the initial angle of the vehicle and Δx indicates the moving distance of the vehicle from the initial position. To verify the accuracy of the theoretical analysis, simulation results are also presented in Figure 5a for comparison, showing that the theoretical analysis aligns well with the simulation results. Figure 5a shows that a greater moving distance of the vehicle results in a higher SER. This increase is attributed to the significant change in the relative position of the vehicle to the RSU post-movement, and the continued use of the initial position’s CSI for decoding leads to degraded SER performance. Moreover, the SER varies with different initial positions, as depicted in Figure 5a. To elucidate the impact of various initial positions on the SER, Figure 5b presents the relationship between the theoretical and simulation SERs at initial positions $\theta_{0,0}$ = −40°, 0°, and 40°. At the initial position, all three SERs are identical because they utilize accurate channel information. When the vehicle’s moving distance is less than approximately 400 m, the SER for an initial position of $\theta_{0,0}$ = 0° is the lowest, the SER for $\theta_{0,0}$ = −40° is intermediate, and the SER for $\theta_{0,0}$ = 40° is the highest. This variation is due to the different changes in the vehicle to RSU distance as the vehicle moves, affecting the relative positions and channel correlations differently. The correlation is highest for $\theta_{0,0}$ = 0°, intermediate for $\theta_{0,0}$ = −40°, and lowest for $\theta_{0,0}$ = 40°. For vehicle moving distances greater than about 400 m, the initial position of $\theta_{0,0}$ = −40° experiences the smallest change in distance and relative position to the RSU, resulting in the smallest change in channel correlation and thus, the lowest SER. The SER for an initial position of $\theta_{0,0}$ = 0° is intermediate, and for $\theta_{0,0}$ = 40°, it is the highest. This is because the channel correlation for $\theta_{0,0}$ = 0° is greater than that for $\theta_{0,0}$ = 40°.

Figure 6 illustrates the variation in the DL communication SER with the initial position of the vehicle and the distance between the vehicle and the RSU in the curved path scenario at SNR = 12 dB. As observed in Figure 6a, the theoretical analysis aligns with the simulation results, validating the accuracy of the theoretical analysis. In the curved path scenario, the focus is on the vehicle’s traversal of the path, with the vehicle’s initial position situated below the RSU. Figure 6a shows that a greater change in the distance between the vehicle and the RSU corresponds to a higher SER. This is attributed to the significant alteration in the vehicle’s relative position to the RSU following movement, and the continued use of the initial position’s CSI for decoding leads to a degradation in SER performance. Figure 6b presents the relationship between the theoretical and simulation SERs at the vehicle’s initial positions set at $\theta_{0,0}$ = −20°, −15°, and −5°. As depicted in Figure 6b, the three SERs are identical at the initial position because of the utilization of precise channel information in the communication process. During the vehicle’s movement, the SER for an initial position of $\theta_{0,0}$ = −5° is the lowest, the SER for $\theta_{0,0}$ = −15° is intermediate, and the SER for $\theta_{0,0}$ = −20° is the highest. This variation occurs because as the vehicle’s moving distance changes, the distance from the vehicle to the RSU and their relative positions change differently, affecting the channel correlation. The correlation is highest for $\theta_{0,0}$ = −5°, intermediate for $\theta_{0,0}$ = −15°, and lowest for $\theta_{0,0}$ = −20°.

In the aforementioned content, we establish a theoretical relationship between the SER of communication and the motion status of the vehicle. This relationship serves as a guide for designing a more efficient communication frame structure. By comparing with simulated data, the accuracy of the theoretical analysis was validated. However, because of limitations in experimental conditions, the aforementioned scheme has not yet been tested in a real V2I environment. We are accelerating the development of a practical testing environment. This will verify how well the scheme discussed in this paper corresponds to real-world conditions.

Based on the above analysis, it is understood that if the relative position between the RSU and the vehicle changes significantly, the channel correlation deteriorates, resulting in an increased SER. Conversely, if the relative position change is minimal, the SER is lowered. Consequently, by establishing a communication SER threshold, the conditions that necessitate re-estimation of the channel can be determined. This approach reduces the frequency of channel estimations and enhances communication efficiency. The following section describes the design scheme of the adaptive frame structure for sensing-assisted DL communication proposed in this paper.

### 3.2. Communication Adaptive Frame Structure Design

The design scheme of the adaptive frame structure for sensing-assisted DL communication proposed in this paper assumes that the vehicle acquires CSI through initial channel estimation and then foregoes further channel estimation during subsequent movement, instead relying on the initial position’s CSI and the sensing capabilities to complete DL communication. Throughout this process, the RSU conducts sensing signal processing using the echo of the communication signal to obtain the vehicle’s distance, angle, and other kinematic parameters during its movement. It then calculates the moving vehicle’s SER using Equation (21). If the computed SER reaches the preset threshold, it indicates that the CSI utilized by the vehicle for demodulation is due for an update. At such junctures, the RSU sends DL pilots, prompting the vehicle to perform channel estimation again and to proceed with the aforementioned process.

**Figure 7:**
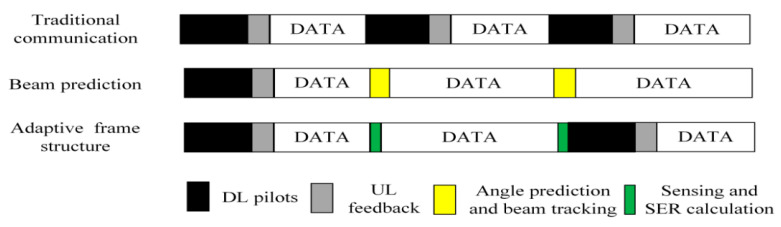
Frame structures for DL communication.

The design concept of the adaptive frame structure is predicated on the RSU employing sensing to acquire the vehicle’s motion parameters. It leverages the theoretical correlation between the SER and these motion parameters, in conjunction with the communication SER requirements, to determine whether to incorporate DL pilots and UL CSI feedback in the subsequent frame. This approach contrasts with traditional communication protocol frame structures, which include pilots only when significant channel variations occur. This strategy can reduce the consumption of time and frequency resources by channel estimation within the frame structure, thereby diminishing the communication overhead. The adaptive frame structure proposed for sensing-assisted DL communication is depicted in Figure 7.

Figure 7 presents the traditional communication protocol (top), the beam prediction-based structure (middle) [32], and the DL communication frame structure of the sensing-assisted adaptive frame structure proposed in this paper (bottom). The traditional communication protocol frame structure necessitates frequent feedback between the RSU and the vehicle to maintain the desired communication SER performance. In contrast to the traditional approach, the beam prediction-based frame structure enables the RSU to conduct beam prediction based on the vehicle’s motion parameters sensing, thus eliminating information exchanges with the vehicle and reducing the beam tracking overhead during communication. However, the vehicle at the receiving end cannot determine the beam’s alteration during decoding, and the channel’s fluctuation as the vehicle moves can result in an increase in the communication SER. Building upon this, the design scheme of the adaptive frame structure for sensing-assisted communication proposed herein considers the impact of beam alignment shifts and channel fading on the SER during the vehicle’s movement. When the SER degradation due to channel changes surpasses a predefined threshold, the system initiates another round of DL channel estimation.

## 4. Simulation and Analysis of Results

This section conducts a comparative analysis of the DL communication performance across three distinct schemes including the following: the traditional communication protocol frame structure, the beam prediction-based frame structure, and the sensing-assisted adaptive frame structure introduced in this paper. Performance is evaluated using metrics such as the SER and system throughput. In the simulation, the vehicle’s speed is maintained at a constant rate. The carrier frequency $f_{c}$ = 6 GHz, and the subcarrier interval Δf = 30 kHz. The OFDM symbol number $M_{s}$ = 14, and the subcarrier number $N_{c}$ = 1680. The antenna interval is set to half the wavelength. The transmitter antenna array size of the RSU is $P_{t}\times Q_{t}$ = 8 × 8. Generally, throughput is calculated by subtracting the overhead and transmission error from the total number of transmitted symbols [43]. The system throughput is defined as follows:

(22) $$Throught(Mbps)=10^{-6}\times \left(P_{t}\times Q_{t}\times Q_{m}\times \frac{N_{sou}\times N_{c}}{T_{s}}\times \left(1-P_{e}-OH\right)\right)$$

where $Q_{m}$, $N_{sou}$, and *OH* denote the modulation order, the actual number of transmitted data resource blocks, and the overhead percentage, respectively, with all other parameters defined as in the previous section.

**Figure 8:**
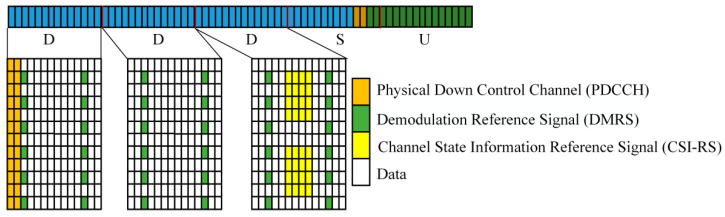
Frame structure for DDDSU.

The traditional protocol employs the “DDDSU” frame structure typical of the V2I scenario, where “D”, “S”, and “U” stand for DL, Special, and UL time slots, respectively [44]. The RSU transmits according to a cycle of five time slots, as depicted in Figure 8.

In the design scheme of the sensing-assisted adaptive frame structure, when channel estimation is not required, CSI-RS is omitted from the frame structure shown in Figure 8, and its time and frequency resources are allocated to actual DL data transmission. Given that there are a total of 504 resource units in one cycle of DL time slots, and considering that CSI-RS occupies 32 resource units, the overhead reduction achieved by the scheme proposed in this paper is calculated to be 32/(504) = 6.35%.

**Figure 9:**
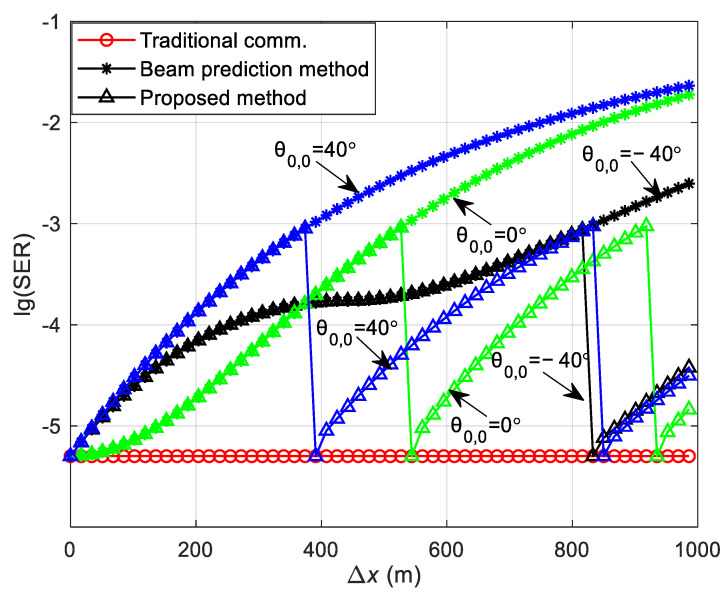
The SER of DL communication in the straight path scenario.

Figure 9 illustrates the variation in the DL communication SER for the three frame structures with the initial position and the moving distance of the vehicle at initial positions of $\theta_{0,0}$ = −40°, 0°, and 40° in the straight path scenario with SNR = 12 dB. Figure 9 shows that at the onset of vehicle movement, the communication SER of the traditional communication protocol frame structure (denoted by circular symbols) is lower than that of the other two structures, and the SER remains consistent across different initial positions. This uniformity is attributed to the periodic channel estimation performed during vehicle movement in the traditional communication protocol frame structure, which ensures that each frame utilizes the precise CSI for information demodulation, resulting in a consistent SER at varying starting positions. The SER of the beam prediction-based frame structure (marked by star symbols) deteriorates as the vehicle’s traveled distance increases because it does not have channel estimation during movement, relying instead on the initial position’s CSI to facilitate the communication process. The SER of the sensing-assisted adaptive frame structure proposed in this paper (indicated by triangular symbols) mirrors that of the beam prediction-based scheme when the movement distance is minimal. However, as the distance grows and the SER exceeds a preset threshold of 1 × 10^−3^, the proposed method performs channel estimation again, aligning its SER with that of the traditional communication protocol frame structure, which outperforms the beam prediction-based frame structure. Furthermore, comparing the vehicle at $\theta_{0,0}$ = 40° with those at $\theta_{0,0}$ = −40° and $\theta_{0,0}$ = 0°, the SER reaches the threshold after a shorter movement distance at $\theta_{0,0}$ = 40°. This is due to the more rapid change in the distance between the vehicle and the RSU at $\theta_{0,0}$ = 40°, leading to a swifter alteration in channel correlation and a more rapid SER increase, thus reaching the threshold sooner. Conversely, at $\theta_{0,0}$ = −40°, the SER reaches the threshold after a longer distance has been traveled because the change in distance between the vehicle and the RSU first decreases and then increases, resulting in smaller channel variations over an extended distance and a slower SER increase.

**Figure 10:**
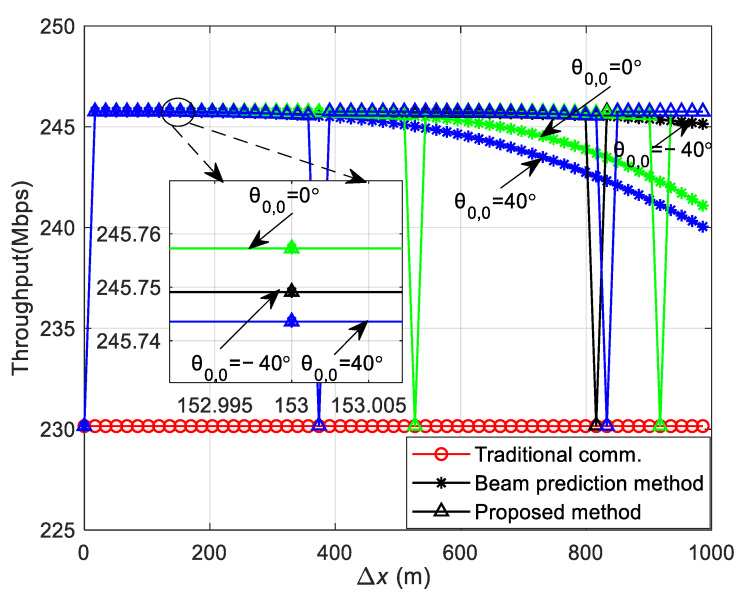
The throughput of DL communication in the straight path scenario.

Figure 10 presents the variation curves of communication throughput for the three frame structures in relation to the vehicle’s initial position and moving distance, with the vehicle’s initial position set at $\theta_{0,0}$ = −40°, 0°, and 40° in the straight path scenario at SNR = 12 dB. It is evident in Figure 10 that when the vehicle commences movement from different initial positions, the communication throughput of the beam prediction-based frame structure exceeds that of the traditional communication protocol frame structure and remains largely constant within a specific distance range, after which it progressively diminishes as the vehicle continues to move further. This phenomenon occurs because the time and frequency resources that would be allocated to channel estimation in the beam prediction-based frame structure are instead utilized for actual data transmission, enhancing the communication throughput. However, as the vehicle’s movement persists, the SER of the beam prediction-based frame structure keeps rising, leading to a decrease in communication throughput. Upon initial movement, a local zoom of Figure 10 reveals that the throughput at $\theta_{0,0}$ = 0° is the highest, at $\theta_{0,0}$ = −40° it is intermediate, and at $\theta_{0,0}$ = 40° it is the lowest. As the vehicle proceeds with its movement, the situation reverses: the throughput at $\theta_{0,0}$ = −40° becomes the highest, at $\theta_{0,0}$ = 0°, it is in the middle, and at $\theta_{0,0}$ = 40°, it is the lowest, mirroring the SER changes observed in Figure 9. The throughput of the sensing-assisted adaptive frame structure proposed in this paper matches that of the beam prediction-based frame structure when the vehicle’s moving distance is minimal. Once the moving distance increases and the SER surpasses the preset threshold of 1 × 10^−3^, the proposed method performs channel estimation again, achieving throughput on par with traditional communication protocol frame structure and thus, outperforming the beam prediction-based frame structure during the vehicle’s movement. Furthermore, at $\theta_{0,0}$ = 40° compared with $\theta_{0,0}$ = −40° and $\theta_{0,0}$ = 0°, the vehicle moves a shorter distance before channel estimation needs to be performed again to match the throughput of the traditional communication protocol frame structure. This is because the SER reaches the threshold value after a shorter movement distance at $\theta_{0,0}$ = 40°. Conversely, at $\theta_{0,0}$ = −40°, channel estimation is performed again only after the vehicle has traversed a longer distance, as the corresponding SER reaches the threshold value later.

**Figure 11:**
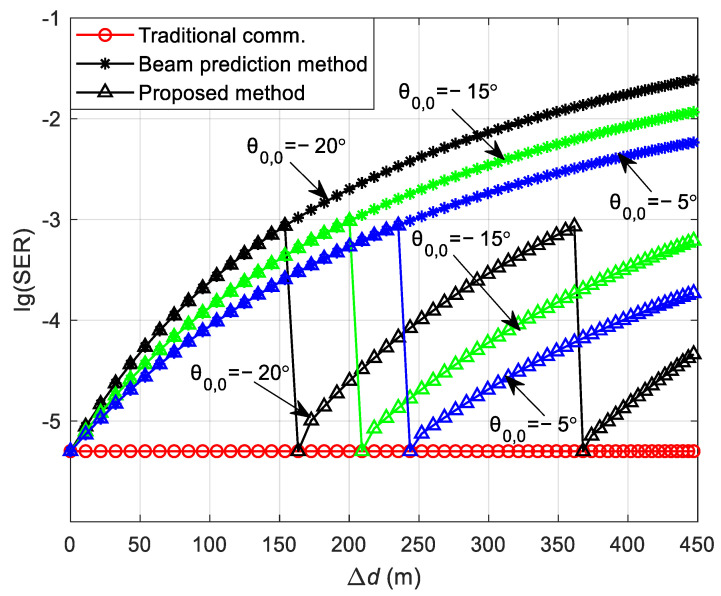
The SER of DL communication in the curved path scenario.

Figure 11 illustrates the variation in the DL communication SER with the initial position of the vehicle and the distance between the vehicle and the RSU for the curved path scenario, with the vehicle’s initial positions set at $\theta_{0,0}$ = −20°, −15°, and −5°, and transmit SNR = 12 dB. It is observable in Figure 11 that the communication SER under the traditional communication protocol frame structure is lower than that of the other two methods, and the SER remains consistent across different initial positions. The SER of the beam prediction-based frame structure progressively deteriorates and exceeds that of the traditional communication protocol frame structure. This degradation is attributed to the absence of channel estimation during the vehicle’s movement, relying instead on the initial position’s CSI to complete the communication process. The SER of the sensing-assisted adaptive frame structure proposed in this paper matches that of the beam prediction-based frame structure when the vehicle’s moving distance is minimal. However, as the moving distance increases and the SER surpasses the preset threshold of 1 × 10^−3^, the proposed method performs channel estimation again, resulting in a SER comparable to that of the traditional communication protocol frame structure, which outperforms the beam prediction-based frame structure. Furthermore, at an initial position of $\theta_{0,0}$ = −20° compared with $\theta_{0,0}$ = −15° and $\theta_{0,0}$ = −5°, the SER reaches the threshold after a shorter vehicle movement distance. This is because, prior to reaching the SER threshold, the distance between the vehicle and the RSU changes more rapidly at $\theta_{0,0}$ = −20°, leading to a swifter alteration in channel correlation. Consequently, the SER increases more rapidly at $\theta_{0,0}$ = −20°, reaching the threshold after a shorter movement distance.

**Figure 12:**
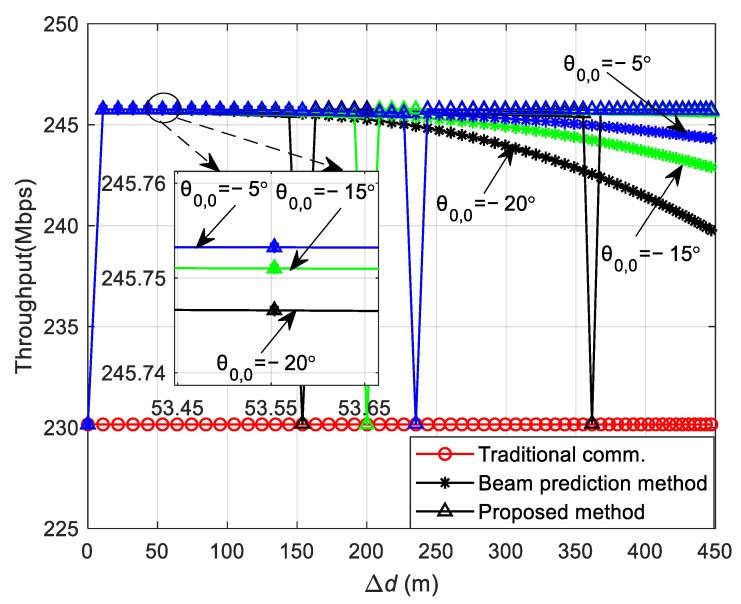
The throughput of DL communication in the curved path scenario.

Figure 12 illustrates the variation in communication throughput relative to the initial position of the vehicle and the distance between the vehicle and the RSU for the three frame structures, with the vehicle’s initial positions in the curved path scenario set at $\theta_{0,0}$ = −20°, −15°, and −5°, and transmit SNR = 12 dB. In Figure 12, it is evident that when the vehicle moves from various initial positions, the communication throughput of the beam prediction-based frame structure initially surpasses that of the traditional communication protocol frame structure and remains relatively stable within a certain distance range. However, as the vehicle persists in moving, the throughput gradually declines. This occurs because the time and frequency resources that would otherwise be allocated to channel estimation in the beam prediction-based frame structure are instead utilized for actual data transmission, enhancing the communication throughput. Nonetheless, as the vehicle continues to move, the SER leads to a marked decrease in communication throughput. Upon initial movement, a local zoom of Figure 12 reveals that at $\theta_{0,0}$ = −5°, the throughput is the highest; at $\theta_{0,0}$ = −15°, it is intermediate; and at $\theta_{0,0}$ = −20°, it is the lowest, correlating with the SER changes observed in Figure 11. The throughput of the sensing-assisted adaptive frame structure proposed in this paper mirrors that of the beam prediction-based frame structure when the vehicle’s moving distance is minimal. Once the moving distance extends and the SER exceeds the preset threshold of 1 × 10^−3^, the proposed method performs channel estimation again, aligning the throughput with that of the traditional communication protocol frame structure and outperforming the beam prediction-based frame structure during the vehicle’s movement. Furthermore, at $\theta_{0,0}$ = −20° compared with $\theta_{0,0}$ = −15° and $\theta_{0,0}$ = −5°, the vehicle moves a shorter distance before channel estimation needs to be performed again to achieve throughput equivalent to the traditional communication protocol frame structure. This is due to the SER reaching the threshold value more rapidly after a shorter movement distance at $\theta_{0,0}$ = −20°. Conversely, at $\theta_{0,0}$ = −15° and $\theta_{0,0}$ = −5°, the vehicle can move a greater distance, as the corresponding SER threshold is attained over a longer distance, reflecting a slower rate of SER increase.

## 5. Conclusions

This paper introduces a design approach for a communication adaptive frame structure that leverages sensing assistance to address the issues of frequent channel estimation and excessive communication overhead in high-speed mobile DL communication scenarios. The RSU utilizes sensing to ascertain the vehicle’s motion state, and in conjunction with the initial CSI, establishes the theoretical relationship between the communication SER and the vehicle’s motion state. The RSU performs real-time estimation of the communication SER based on the vehicle’s movement distance during travel. If the SER surpasses a predefined threshold, the RSU incorporates pilots into the transmit signal’s frame structure to perform channel estimation again. If the real-time SER during the vehicle’s movement remains below the designated threshold, the transmission of pilots for channel estimation becomes unnecessary, thereby reducing the frequency of channel estimation events, minimizing the time and frequency resources consumed by channel estimation within the frame structure, and enhancing communication efficiency. Simulation results show that the communication throughput of the proposed sensing-assisted adaptive frame structure can be improved by up to 6% while still maintaining the SER within acceptable limits.

## Data Availability

Any data or method mentioned in this article can be used or referenced by other authors.

## References

[1] Deepender M., Shrivastava U., Verma J.K. A study on 5G technology and its applications in telecommunications. Proceedings of the 2021 International Conference on Computational Performance Evaluation (ComPE).

[2] Liu G., Huang Y., Li N., Dong J., Jin J., Wang Q., Li N. (2020). Vision, requirements and network architecture of 6G mobile network beyond 2030. China Commun..

[3] Ghafoo K.Z., Kong L., Rawat D.B., Hosseini E., Sadiq A.S. (2019). Quality of service aware routing protocol in software-defined internet of vehicles. IEEE Internet Things J..

[4] Kaiwartya O., Abdullah A., Cao Y., Altameem A., Prasad M., Lin C.T., Liu X. (2016). Internet of vehicles: Motivation, layered architecture, network model, challenges, and future aspects. IEEE Access.

[5] Zhong Y., Bi T., Wang J., Zeng J., Huang Y., Jiang T., Wu Q., Wu S. (2022). Empowering the V2X network by integrated sensing and communications: Background, design, advances, and opportunities. IEEE Netw..

[6] Yuan W.J., Li S.Y., Xiang L., Ng D.W.K. (2020). Distributed estimation framework for beyond 5G intelligent vehicular networks. IEEE Open J. Veh. Technol..

[7] Siegel J.E., Erb D.C., Sarma S.E. (2017). A survey of the connected vehicle landscape architectures, enabling technologies, applications, and development areas. IEEE Trans. Intell. Transp. Syst..

[8] Ozkaptan C.D., Zhu H., Ekici E., Altintas O. Software-Defined MIMO OFDM joint radar-communication platform with fully digital mmWave architecture. Proceedings of the 2023 IEEE 3rd International Symposium on Joint Communications & Sensing (JC&S).

[9] Wei Z., Wang Y., Ma L., Yang S., Feng Z., Pan C., Zhang Q., Wang Y., Wu H., Zhang P. (2023). 5G PRS-Based sensing: A sensing reference signal approach for joint sensing and communication system. IEEE Trans. Veh. Technol..

[10] Zhao Q., Tang A., Wang X. (2023). Reference signal design and power optimization for energy-efficient 5G V2X integrated sensing and communications. IEEE Trans. Green Commun. Netw..

[11] Khan U., Jameel F., Kumar N., Jäntti R., Guizani M. (2021). Backscatter-Enabled efficient V2X communication with non-orthogonal multiple access. IEEE Trans. Veh. Technol..

[12] Chattopadhyay R., Tham C.K. (2023). Joint sensing and processing resource allocation in vehicular Ad-Hoc networks. IEEE Trans. Intell. Veh..

[13] Bartoletti S., Decarli N., Masini B.M. Sidelink 5G-V2X for integrated sensing and communication: The impact of resource allocation. Proceedings of the 2022 IEEE International Conference on Communications Workshops (ICC Workshops).

[14] Decarli N., Bartoletti S., Bazzi A., Stirling-Gallacher R.A., Masini B.M. (2024). Performance characterization of joint communication and sensing with beyond 5G NR-V2X sidelink. IEEE Trans. Veh. Technol..

[15] Wang Y., Liang W., Li L., Zhang J., Angelopoulos C.M. Intelligent predictive beamforming for integrated sensing and communication based vehicular-to-infrastructure systems. Proceedings of the 2023 IEEE Globecom Workshops (GC Wkshps).

[16] Lin Y., Ke F., Chen M., Qin M., Lee Y.L., Li D. Joint communication, sensing and computing for V2I networks. Proceedings of the 2023 IEEE 98th Vehicular Technology Conference (VTC2023-Fall).

[17] Fan W., Su Y., Liu J., Li S., Huang W., Wu F., Liu Y. (2023). Joint task offloading and resource allocation for vehicular edge computing based on V2I and V2V models. IEEE Trans. Intell. Transp. Syst..

[18] Ghafoor K.Z., Kong L., Zeadally S., Sadiq A.S., Epiphaniou G., Hammoudeh M., Bashir A.K., Mumtaz S. (2020). Millimeter-wave communication for internet of vehicles: Status challenges and perspectives. IEEE Internet Things.

[19] Xiao Z., Qi C., Nie J. (2023). Beam tracking based on variable step beam for millimeter wave massive MIMO. IEEE Commun. Lett..

[20] Chen K., Qi C., Wang C.X., Li G.Y. (2024). Beam training and tracking for extremely large-scale MIMO communications. IEEE Trans. Wirel. Commun..

[21] Yang S., Ma J., Zhang S., Li H. Beam prediction for mmWave massive MIMO using adjustable feature fusion learning. Proceedings of the 2022 IEEE 95th Vehicular Technology Conference: (VTC2022-Spring).

[22] Gonzalez-Prelcic N., Mendez-Rial R., Heath R.W. Radar aided beam alignment in mmwave V2I communications supporting antenna diversity. Proceedings of the Information Theory and Applications Workshop (ITA).

[23] Heath R.W., Gonzlez-Prelcic N., Rangan S., Roh W., Sayeed A.M. (2016). An overview of signal processing techniques for millimeter wave MIMO systems. IEEE J. Sel. Top. Signal Process..

[24] Zhang D., Li A., Shirvanimoghaddam M., Cheng P., Li Y., Vucetic B. (2019). Codebook-Based training beam sequence design for millimeter-wave tracking systems. IEEE Trans. Wirel. Commun..

[25] Shaham S., Ding M., Kokshoorn M., Lin Z., Dang S., Abbas R. (2019). Fast channel estimation and beam tracking for millimeter wave vehicular communications. IEEE Access.

[26] Liu F., Masouros C. (2020). A tutorial on joint radar and communication transmission for vehicular networks-Part I: Background and fundamentals. IEEE Commun. Lett..

[27] Kuutti S., Fallah S., Katsaros K., Dianati M., Mccullough F., Mouzakitis A. (2018). A survey of the state-of-the-art localization techniques and their potentials for autonomous vehicle applications. IEEE Internet Things J..

[28] Hyun S.-H., Song J., Kim K., Lee J.H., Kim S.C. (2022). Adaptive beam design for V2I communications using vehicle tracking with extended kalman filter. IEEE Trans. Veh. Technol..

[29] Du Z., Liu F., Yuan W., Masouros C., Zhang Z., Xia S. (2023). Integrated sensing and communications for V2I networks: Dynamic predictive beamforming for extended vehicle targets. IEEE Trans. Wirel. Commun..

[30] Meng X., Liu F., Masouros C., Yuan W., Zhang Q., Feng Z. (2023). Vehicular connectivity on complex trajectories: Roadway-geometry aware ISAC beam tracking. IEEE Trans. Wirel. Commun..

[31] Yuan W., Liu F., Masouros C., Yuan J., Ng D.W.K., González-Prelcic N. (2020). Bayesian predictive beamforming for vehicular networks: A low-overhead joint radar communication approach. IEEE Trans. Wirel. Commun..

[32] Liu F., Yuan W., Masouros C., Yuan J. (2020). Radar-Assisted predictive beamforming for vehicular links: Communication served by sensing. IEEE Trans. Wirel. Commun..

[33] Liu C., Yuan W., Liu S., Liu X., Li H., Ng D.W.K., Li Y. (2022). Learning-Based predictive beamforming for integrated sensing and communication in vehicular networks. IEEE J. Sel. Areas Commun..

[34] Dosovitskiy A., Ros G., Codevilla F., Lopez A., Koltun V. CARLA: An open urban driving simulator. Proceedings of the 1st Conference on Robot Learning (CoRL).

[35] Stepanyants V.G., Romanov A.Y. (2024). Influence of realistic perception and surroundings on qualitative results in automated and connected vehicle simulation. IEEE Access.

[36] Lopez P.A., Behrisch M., Bieker-Walz L., Erdmann J., Flötteröd Y.P., Hilbrich R., Lücken L., Rummel J., Wagner P., Wiessner E. Microscopic traffic simulation using sumo. Proceedings of the 21st IEEE International Conference on Intelligent Transportation Systems.

[37] Xu R., Xiang H., Han X., Xia X., Meng Z., Chen C.J., Correa-Jullian C., Ma J. (2023). The OpenCDA open-source ecosystem for cooperative driving automation research. IEEE Trans. Intell. Veh..

[38] Chen X., Feng Z., Wei Z., Zhang J.A., Yuan X., Zhang P. (2023). Concurrent downlink and uplink joint communication and sensing for 6G networks. IEEE Trans. Veh. Technol..

[39] Chen X., Feng Z., Zhang J.A., Wei Z., Yuan X., Zhang P., Peng J. (2024). Downlink and uplink cooperative joint communication and sensing. IEEE Trans. Veh. Technol..

[40] Stepanyants V., Romanov A. An object-oriented approach to a structured description of machine perception and traffic participant interactions in traffic scenarios. Proceedings of the 2022 IEEE 7th International Conference on Intelligent Transportation Engineering (ICITE).

[41] Scholtes M., Westhofen L., Turner L.R., Lotto K., Schuldes M., Weber H., Wagener N., Neurohr C., Bollmann M.H., Körtke F. (2021). 6-Layer model for a structured description and categorization of urban traffic and environment. IEEE Access.

[42] Basar E. (2020). Reconfigurable intelligent surface-based index modulation: A new beyond MIMO paradigm for 6G. IEEE Trans. Commun..

[43] (2018). User Equipment Radio Access Capabilities. 3rd Generation Partnership Project.

[44] (2022). Physical Channels and Modulation.

